# ^68^Ga-radiolabeled fluorescent dye for potential non-invasive multimodal imaging of subarachnoid hemorrhage

**DOI:** 10.1186/s41181-025-00348-5

**Published:** 2025-07-09

**Authors:** Jona Wilhelm Gerhards, Laura Schäfer, Daniel Kang, Ute Lindauer, Susanne Lütje, Felix Manuel Mottaghy, Tobias Schmidt, Andreas Theodor Josef Vogg

**Affiliations:** 1 Department of Nuclear Medicine, RWTH Aachen University Hospital, Pauwelsstraße 30, 52074 Aachen, Germany; 2 Translational Neurosurgery and Neurobiology, Department of Neurosurgery, RWTH Aachen University Hospital, 52074 Aachen, Germany

**Keywords:** Stroke, Subarachnoid hemorrhage, Gallium-68, Alexa Fluor™ 594, NODA-GA-NHS ester, Positron emission tomography, Multimodal imaging

## Abstract

**Background:**

Aneurysmal subarachnoid hemorrhage (aSAH) is a distinct type of stroke, primarily caused by the rupture of a brain aneurysm. The underlying mechanisms of aSAH remain incompletely understood, prompting ongoing research in this area. Recent investigations into the perivascular system revealed a distribution disturbance of the dye Alexa Fluor™ 594 during measurements. To further investigate this distribution anomaly, it is proposed to label the dye with a radionuclide for biokinetic tracking in rats by means of positron emission tomography for enhanced imaging and analysis.

**Results:**

The fluorescent dye Alexa Fluor™ 594 after chelator conjugation was successfully labeled with the positron-emitting radionuclide ^68^Ga(III) in a *no-carrier-added* form. Initially, the NODA-GA-NHS ester was employed to react with the amino group of Alexa Fluor™ 594 1,5-diaminopentane, facilitating subsequent radiolabeling with ^68^Ga. The formation of the Alexa Fluor™ 594-chelator conjugate, as well as the radiolabeling, were investigated as a function of reaction time and temperature. For potential animal experiments, it was necessary to increase the reaction temperature from room temperature to 80 °C to optimize the reaction conditions, given the short half-life of ^68^Ga. Optimal labeling conditions were established, achieving a radiochemical yield of > 85%. Separation and purification of n.c.a. [^68^Ga]Ga-NODA-GA-Alexa Fluor™ 594 were conducted, with impurities remaining below 3%.

**Conclusions:**

This experimental approach successfully yields the desired radiolabeled dye, which is now available for animal studies, potentially offering enhanced insight into the mechanisms of aSAH.

## Background

Aneurysmal subarachnoid hemorrhage (aSAH) is a critical neurological condition caused by bleeding into the subarachnoid space due to the rupture of intracranial aneurysm (Maher et al. 2020). This type of stroke poses significant clinical challenges, as aSAH is associated with high morbidity and mortality rates (van Amerongen et al. 2014), resulting from both primary and secondary pathomechanisms (Lawton and Vates 2017). Early brain injury occurs immediately after aneurysmal rupture, while secondary processes such as cortical spreading depolarization, neuronal cell death, and blood brain barrier breakdown may further contribute to disease progression and secondary deterioration. As a result, aSAH remains a subject of intense research aimed at elucidating its pathophysiological mechanisms. Despite advancements in understanding the early pathophysiological changes of aSAH (Huang and van Gelder 2002), the underlying biological processes and the role of the perivascular system in the progression of this condition are still not fully understood. Recent studies have highlighted a notable disturbance in the distribution of the fluorescent dye Alexa Fluor™ 594 within the perivascular space, prompting further investigation into this anomaly as a potential indicator of altered perivascular dynamics following aSAH. Blood products and clot formation after aSAH impede the glymphatic flow, delaying the removal of neurotoxic substances and promoting tissue damage (Golanov, et al. 2018).

To deepen the understanding of the mechanisms contributing to aSAH, innovative imaging techniques capable of visualizing perivascular flow dynamics are needed. Positron emission tomography (PET), a powerful non-invasive imaging modality, holds promise for providing real-time insights into biological processes at the molecular level. However, its application to glymphatic studies requires suitable radiotracers that can track interstitial and perivascular transport.

**Fig. 1:**
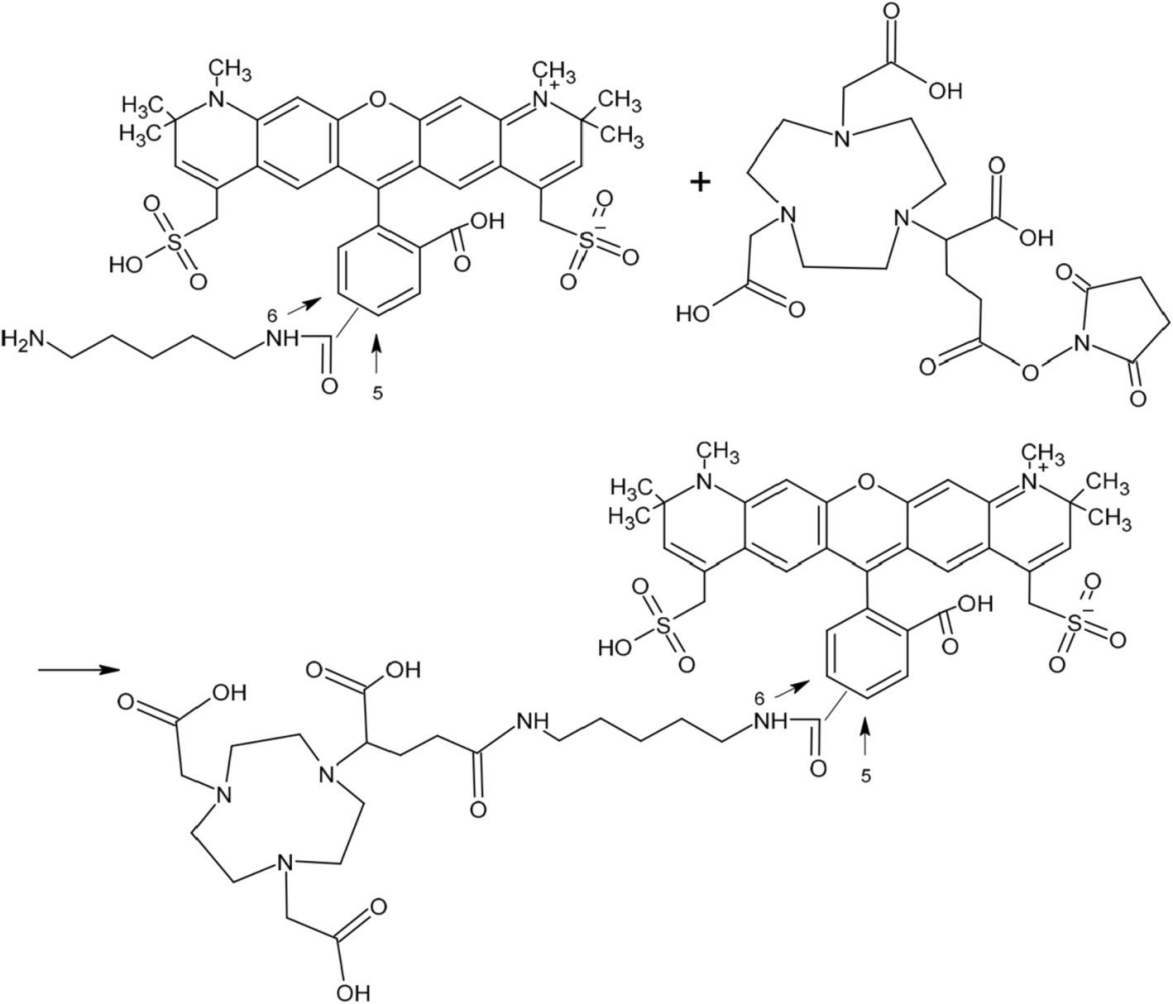
Reaction scheme of Alexa Fluor™ 594 1,5-diaminopentane (mixture of constitution isomers regarding the linker substitution at the benzoic ring) and NODA-GA-NHS ester (racemate). Substitution of the linker at the benzoic moiety is random, resulting in dye isomers

In this study, we present a novel approach to label Alexa Fluor 594™ 1,5-diaminopentane, a dye previously utilized for fluorescence imaging, with the positron-emitting radionuclide Gallium-68 (^68^Ga) (Fig. 1). By employing a *no-carrier-added* radiolabeling technique, we aimed at optimizing the conjugation of the dye with ^68^Ga by means of an appropriate chelator, facilitating its application in animal models for PET imaging. The radiolabeled compound, [^68^Ga]Ga-NODA-GA-Alexa Fluor™ 594, is designed to trace the perivascular distribution pathways, thereby enabling the assessment of glymphatic system integrity and function following aSAH. By visualizing alterations in tracer transport dynamics, it becomes possible to identify impaired clearance and regional disruptions of the glymphatic system.

Herein, we detail the methodology for conjugation of Alexa Fluor™ 594 with the chelator 2,2'-(7-(1-carboxy-4-((2,5-dioxopyrrolidin-1-yl)oxy)−4-oxobutyl)−1,4,7-triazonane-1,4-diyl)diacetic acid (NODA-GA-NHS ester), followed by the radiolabeling with ^68^Ga, including the optimization of reaction conditions to achieve high radiochemical yields. Our findings not only establish a reliable procedure for radiolabeling but also pave the way for future investigations into the dynamics of aSAH. This work provides a novel imaging tool for investigating how aSAH affects glymphatic function, with the ultimate goal of better understanding the disease's pathophysiology and identifying potential therapeutic targets aimed at restoring glymphatic flow.

## Results

### Conjugation of the NODA-GA-NHS ester to Alexa Fluor™ 594 1,5-diaminopentane

**Fig. 2:**
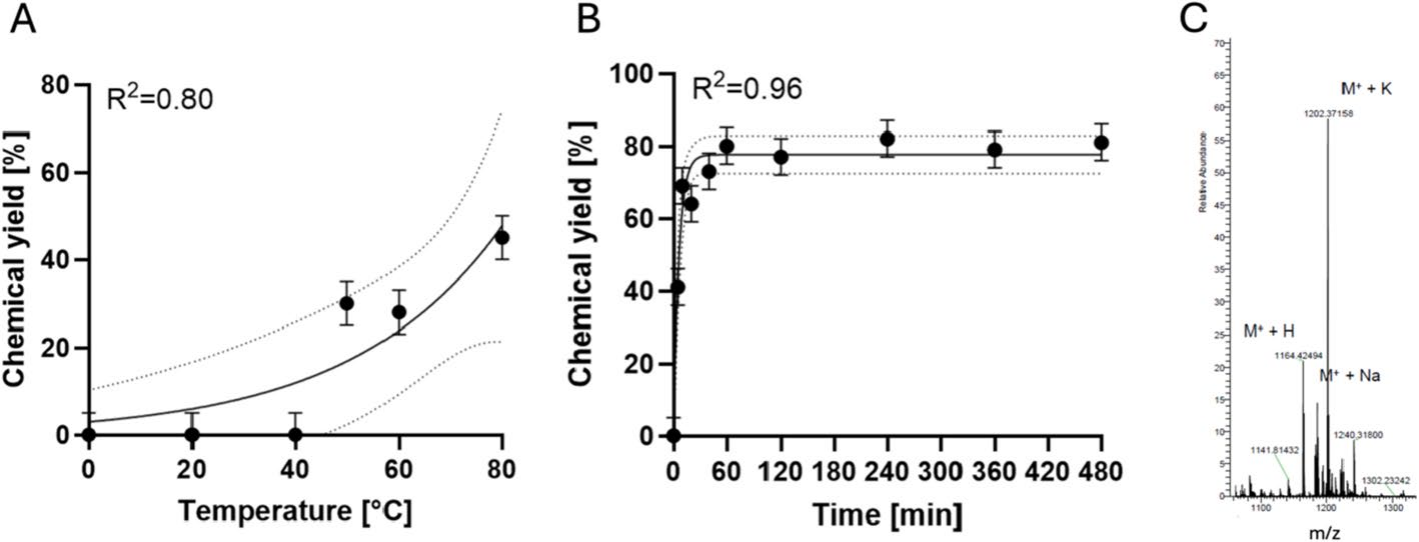
Synthesis and characterization of NODA-GA-Alexa Fluor™ 594 conjugate. **A** Optimization of reaction temperature. Reaction of NODA-GA NHS ester (10 µl, 0.02 M in MeCN) with Alexa Fluor™ 594 1,5-diaminopentane (1 µl, 0.01 M in dimethyl sulfoxide (DMSO)), pyridine (2 µl) and MeCN (5 µl); molar ratio chelator:dye was 20:1. Reaction over 30 min in a 0.1 ml Eppendorf Fast PCR tube. Dotted lines indicating 95% confidence intervals. **B** Effect of reaction time on the yield. Molar ratio chelator:dye again was 20:1. Reaction components: NODA-GA NHS ester (2 µl in MeCN, 0.1 M), Alexa Fluor™ 1,5-diaminopentane (1 µl in DMSO), pyridine (2 µl) and MeCN (13 µl) at 80 °C in 0.1 ml Eppendorf Fast PCR Tubes. Dotted lines indicating 95% confidence intervals. **C** Mass spectrometer analysis of the purified conjugation product of Alexa Fluor™ 594 1,5-diaminopentane and NODA-GA-NHS ester. Only intact mass range 1100–1300 m/z is shown

The first step in the synthesis protocol involved the formation of a stable conjugate between the NODA-GA-NHS ester and Alexa Fluor™ 594 1,5-diaminopentane. This reaction was conducted in acetonitrile (MeCN) rather than in water. Pyridine was used as auxiliary base to facilitate the conjugation rather than triethylamine, as used by Ashhar et al. (Ashhar et al. 2020). Optimization of the reaction parameters focused on temperature, the ratio of chelator to dye, and reaction time. The optimal reaction temperature was found to be 80 °C, yielding nearly 50% chemical yield within 30 min (Fig. 2A), which was limited by the boiling point of MeCN (82 °C).

We found that a chelator-to-dye molar ratio of at least 20:1, combined with an 80 °C reaction temperature, was necessary to achieve sufficient conjugation yields with the dye. Lower ratios (10:1, 5:1) of chelator-to-dye clearly resulted in lower yields. By further increasing the reaction time to one hour, the maximum yield could be increased to 80% at the chosen conditions, as revealed by the kinetics analysis (Fig. 2B). The resulting fluorescent conjugate was purified from excess NODAGA-NHS ester using a pre-conditioned C18 solid-phase extraction cartridge. The nonpolar dye-containing fraction was eluted with ethanol.

Confirmation of successful product formation was achieved through Orbitrap Mass Spectrometry Analysis. The theoretical molecular weight of the product was calculated to be 1163.4 g/mol. The mass spectrometry analysis revealed three significant peaks corresponding to the product (Fig. 2C): m/z 1164.4 (M + H), 1186.4 (M + Na), and 1202.4 (M + K). These peaks are indicative of the expected product, with the observed values representing the conjugation product (M) in addition to a hydrogen atom, sodium ion, and potassium ion, respectively. Indeed, with regard to the clear functional groups amine (dye) and NHS ester (chelator) no other side reactions should occur. As expected, UV absorbance and fluorescence emission spectra of the dye-chelator conjugate showed no peak shift compared to the native dye. The chelator has no quenching properties.

### *Radiolabeling with *^*68*^*Ga*

**Fig. 3:**
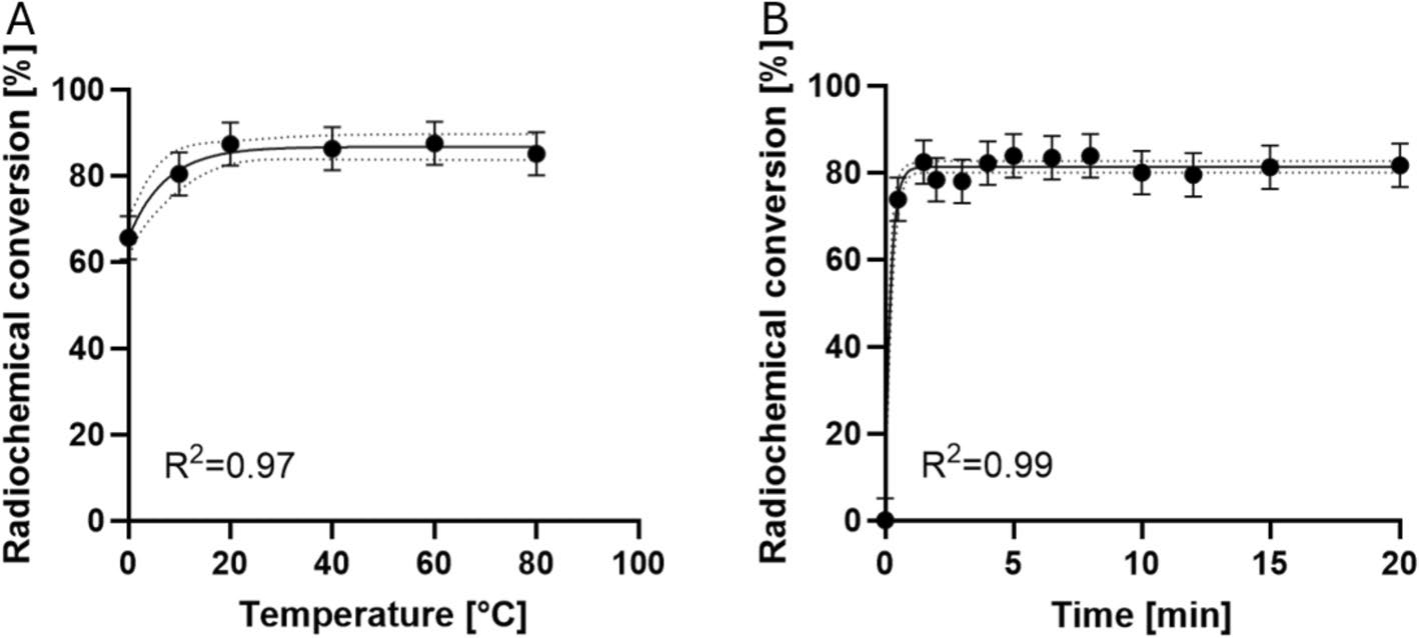
Radiolabeling of NODA-GA-Alexa Fluor™ 594 conjugate with n.c.a. ^68^Ga. **A** RCC as a function of reaction temperature. Reaction of NODA-GA-Alexa Fluor™ 594 conjugate (5 µl, 0.5 mM in ethanol) with n.c.a. [^68^Ga]GaCl_3_ (10 µl in 0.6 M hydrochloric acid (HCl)) in the presence of ammonium acetate buffer (5 µl, 3 M). Reaction time 15 min in a 0.1 ml Eppendorf Fast PCR tube, reaction conjugate conc. was 125 µM. Dotted lines indicating 95% confidence intervals. **B** Effect of reaction time on the RCC. Reaction at room temperature (24 °C) of NODA-GA-Alexa Fluor™ 594 conjugate (15 µl, 0.5 mM in ethanol) with [^68^Ga]GaCl_3_ (10 µl, in 0.6 M HCl)) in the presence of ammonium acetate buffer (15 µl, 3 M) in a 0.1 ml Eppendorf Fast PCR Tube, reaction conjugate concentration was 188 µM. Dotted lines indicating 95% confidence intervals

The second step in the synthesis protocol involved the radiolabeling of the chelator-dye conjugate using ^68^Ga. Reaction conditions were systematically optimized, focusing on conjugate concentration, reaction temperature, and reaction time. It was observed that conjugate concentrations exceeding 125 µM within the reaction volume resulted in radiochemical conversions (RCC) greater than 85%; 50 µM achieved only 70%. Notably, yields did not increase further with higher concentrations of the conjugate. Nevertheless, subsequent optimization reactions were performed using a conjugate concentration of 125 µM or higher to achieve stable optimal labeling. Investigations of the RCC on reaction temperature indicated that yield saturation already occurred at room temperature, reaching 87% (Fig. 3A). In terms of reaction time at room temperature, the maximum RCC of over 80% was achieved already after 2 min (Fig. 3B).

### Upscaling for animal experiments

For subsequent animal experiments, the preparation of the radiotracer was forced to undergo specific modifications to meet the requirements for in vivo applications. Intravenous injections in animal studies necessitate radiotracers with high activity concentrations and injection volumes specifically for mice of less than 5 ml/kg of body weight (Dülsner et al. 2017), which is even more demanding for intrathecal administration [for mouse: 10 µl (Golanov et al. 2018)] as needed for studying perivascular distribution in aSAH. Additionally, it is essential to ensure that the product solution is pure, free from contaminants, and formulated in an ideally isotonic solution with a neutral pH.

**Fig. 4:**
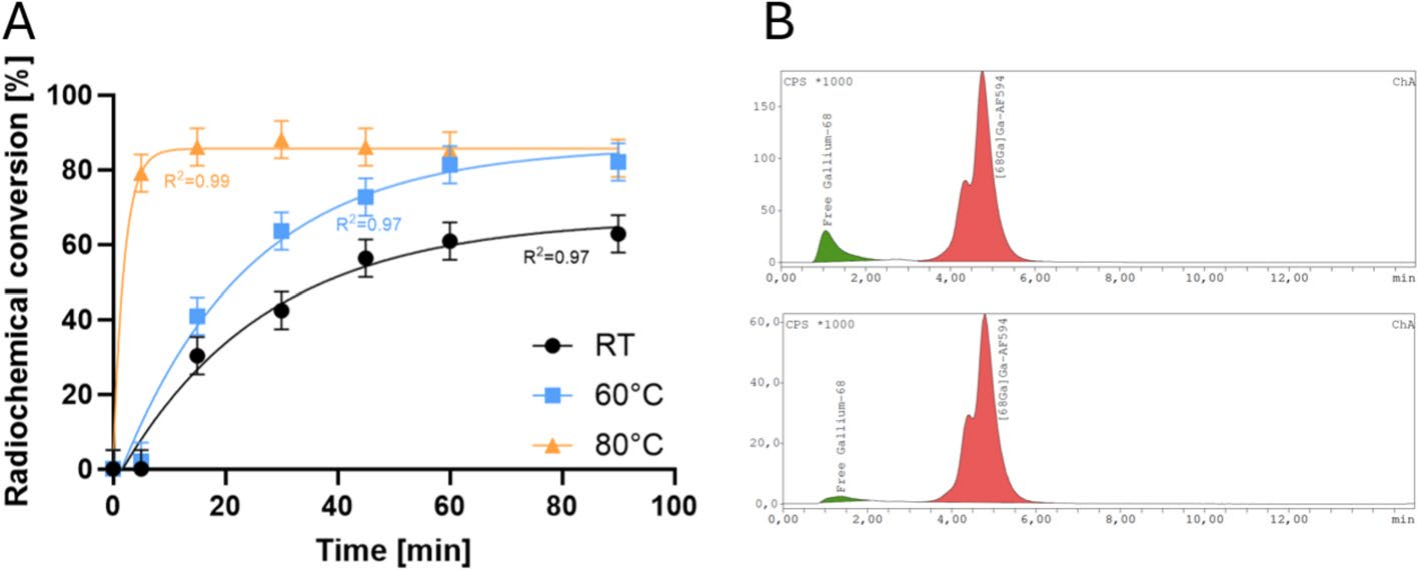
Radiolabeling of NODA-GA-Alexa Fluor™ 594 conjugate with ^68^Ga for animal experiments. **A** Optimization of reaction temperature. Shown is the RCC in dependence on reaction time. Reaction of the NODA-GA-Alexa Fluor™ 594 conjugate (15 µl, 0.5 mM) with n.c.a. [^68^Ga]GaCl_3_ (50 MBq, 100 µl in 0.6 M HCl) in the presence of buffer (10 µl, 6 M ammonium acetate, pH 4.5). Reaction in a 0.1 ml Eppendorf Fast PCR Tube, reaction conjugate concentration was 60 µM. **B** Quality control: High performance liquid chromatography (HPLC) analysis of the product before and after C18 cartridge purification. HPLC settings: Radiodetector: Gamma detection (NaI). A gradient elution method was used (Solvent A: H_2_O/Trifluoroacetic acid (99.9/0.1% v/v); and Solvent B: MeCN) at a flow rate of 1 mL/min and a HPLC affinity C18 column (50 × 3 mm, particle size 2 μm). Within HPLC analyses the dye always shows two distinct isomers

Higher desired ^68^Ga product activities unfortunately imply higher nuclide volumes, thus letting the reaction volume increase. However, due to the high costs for the dye, the precursor concentration could not be adapted accordingly, thus leading to slower reaction kinetics. Hence, at room temperature, the labeling reaction proceeded slowly, resulting in a lower overall yield of 60% after 60 min (Fig. 4A) at a halved conjugate concentration of 60 µM. To achieve rapid radiolabeling, given the short half-life of ^68^Ga (68 min), the labeling reaction temperature was increased to 80 °C. This adjustment led to RCC of 78% after just 5 min, which increased to over 85% after 15 min of reaction time (Fig. 4A). For purification and especially for removal of free ^68^Ga(III), a C18 cartridge was utilized and the organic eluate was evaporated at 80 °C and the residue reconstituted in isotonic sodium chloride. The final product achieved a radiochemical purity of over 97% and was yielded in activities higher than 20 MBq.

## Discussion

In this study, we successfully coupled Alexa Fluor™ 594 1,5-diaminopentane with NODA-GA-NHS ester through amide bond formation to create a conjugate capable of chelating ^68^Ga. The isomers of the dye basically show two distinct fractions of different lipophilicity. This, virtually, did not affect the cerebral fluidic distribution. As a fluid tracer, also the racemic nature of the chosen chelator has no impact. In our approach, we opted for a hydrophobic solvent instead of water based on the methodology outlined by Ashhar et al. (2020), as NHS-esters are prone to hydrolysis over time (Hermanson 2013). Unlike Ashhar et al., who used triethylamine as the base, we used pyridine as auxiliary base with reduced nucleophilicity. This combination proved to be effective, and as a result, no further bases were evaluated.

Given the poor solubility of Alexa Fluor™ 594 in MeCN, DMSO was utilized to prepare the dye stem solution. Conducting the reaction at an elevated temperature of 80 °C necessitated periodic shaking of the reaction tube to condense any evaporated solvent back into the reaction mixture. Attempts to use only DMSO as the solvent to allow higher reaction temperatures and potentially enhance yields resulted in reduced conjugate formation, even at elevated temperatures. Stability experiments confirmed that Alexa Fluor™ 594 remained stable up to 100 °C, indicating that temperature was not a limiting factor for dye integrity.

For the labeling, we followed the methodology established by Ashhar et al. (2020). A favorable reaction pH of 4.5 was determined to yield optimal RCC. This pH was achieved by applying a volume ratio of 2:1 regarding [^68^Ga]GaCl_3_ (in 0.6 M HCl) and ammonium acetate buffer (3 M). Initially, the labeling yield was quite low due to the presence of unreacted NODA-GA-NHS-ester in the reaction mixture. Therefore, it was essential to remove the excess free chelator using a C18 cartridge for conjugate purification. This significantly improved the labeling yields. Notably, we found that when using 125 µM conjugate in the reaction, room temperature was sufficient to achieve high RCC, which is in line with the results of Chand et al. (2020) and Zhao et al. (2025). Other studies, however, typically suggest that higher temperatures are necessary for similar RCC (Haubner et al. 2016; Tsionou et al. 2017; Ashhar et al. 2020). Crucial in this context is, however, the chelator concentration within the reaction volume. We saw a similar need for increasing the reaction temperature during the upscaling of the tracer production process since we could not increase the conjugate concentration accordingly.

For the initial establishment of radiolabeling, small amounts of radioactivity and volumes were sufficient. However, this requirement changed when preparing radiotracer batches for animal experiments. As the reaction volume was scaled up, it became necessary to increase the reaction temperature to facilitate rapid radiolabeling with n.c.a. ^68^Ga. At room temperature, the labeling efficiency did not exceed 60% after more than one hour. In contrast, raising the temperature to 80 °C improved the reaction kinetics and led to significantly better RCC (> 80%) within less than 10 min. This observation aligns with current literature on the radiolabeling of NODA-GA chelator with ^68^Ga (Haubner et al. 2016; Tsionou et al. 2017; Ashhar et al. 2020). Hence, the reaction parameter temperature plays a critical role in optimizing the radiolabeling process, particularly when scaling up for larger production volumes while reducing precursor concentrations.

## Conclusions

This study successfully developed a radiolabeled version of Alexa Fluor™ 594 using the positron-emitting radionuclide ^68^Ga, thus offering a novel approach for in vivo tracking of the dye in animal models. The optimized labeling procedure achieved a high RCC of > 85%, with minimal impurities (< 3%), making it suitable for use in biological imaging. The use of this radiolabeled dye will provide valuable insights into the distribution anomalies observed in the perivascular system during aSAH, offering a deeper understanding of the pathophysiological mechanisms at play.

## Methods

### Materials

The chemicals used were obtained from different manufacturers in varying quality. Ammonium acetate (puriss. p.a.), pyridine (> 99%), trifluoacetic acid (for HPLC) and triethylamine (> 99.5%) were purchased from Sigma Aldrich, MeCN (for DNA analysis), ethanol (EMSURE), sodium bicarbonate (for analysis EMSURE), HCl (Suprapure) and water (Ultrapure) from Merck, benzylamine (AcroSeal > 99.5%) and DMSO (anhydrous 99.7%) from Acros Organics, and Alexa Fluor™ 594 cadavine [Alexa Fluor™ 594 1,5-diaminopentane] from Thermo Fisher Scientific (product A30678, a mixture of substitution isomers regarding the linker). NODA-GA-NHS ester (racemate) was purchased from CheMatech (product C098) and EDTA (pure) from Serva. HPLC running fluids were purchased from Carl Roth (water Rothisolv HPLC Gradient Grade) and from PanReac AppliChem (acetonitrile for UHPLC Super gradient). The argon used was from Linde (Argon 4.8 for spectrometry). The reactions were performed in Eppendorf 1.5 ml Safe-Lock tubes and Eppendorf 0.1 Fast PCR Tube Strips (Polypropylene). N.c.a. [^68^Ga]GaCl_3_ in 0.6 M HCl was supplied by a ^68^Ge/^68^Ga radionuclide generator from iThemba LABS/NRF, South Africa. PS-H cartridges (M size) were obtained from Macherey–Nagel (Düren, Germany). C18 cartridges (130 mg Sorbent, 55–105 µm) from Waters™.

### High performance liquid chromatography (HPLC)

Analytical reversed-phase HPLC was performed using an Knauer HPLC system equipped with a quaternary pump and UV−vis- and radio detector with a NaI-scintillator crystal produced by Raytest. The used HPLC-column was a Chromolith Fast Gradient RP-18e/50*3 mm from Merck. For the mobile phase (A) water, with an addition of 0.1% of trifluoroacetic acid, and (B) pure MeCN were used for performing gradient RP-HPLC analyses.

### Mass spectroscopy

Mass spectroscopy measurement. The used mass spectrometer was an Orbitrap XL from Thermo Fisher Scientific with an ESI-Ionization source.

### Other devices

An activimeter (FNR 102.109) with ISOMED 2010 software version 5.4.6.6, supplied by Nuvia was used for the measurement of activities.

### Conjugation of the NODA-GA-NHS ester to Alexa Fluor™ 594 1,5-diaminopentane

Alexa Fluor™ 594 1,5-diaminopentane was dissolved in DMSO to a concentration of 0.01 M serving as stem solution for all subsequent experiments. The conjugation of NODA-GA-NHS ester to Alexa Fluor™ 594 1,5-diaminopentane followed the protocol outlined by Ashhar et al. (Ashhar, Yusof et al. 2020). To initiate the reaction, 1 µl of Alexa Fluor™ 594 1,5-diaminopentane (0.01 M in DMSO), 2 µl of NODA-GA-NHS ester (0.1 M in MeCN), 2 µl of pyridine, and 5 µl of MeCN were combined and reacted for one hour at 80 °C in a 0.1 ml Fast PCR tube. Product formation and kinetic studies were analyzed using mass spectrometry and HPLC. The chelator was used in excess during the reaction with the dye (molar ratio dye:chelator = 1:20). The excess chelator was then removed using a C18-SPE cartridge. The cartridge was conditioned with 1 ml of ethanol, followed by 10 ml of distilled water. The sample was diluted with 3 ml of water and passed through the cartridge. The cartridge was washed with 7 ml of water to remove excess free chelator, and the nonpolar dye fraction was eluted with 1 ml of ethanol. Product quality control: Incomplete derivatization of the native dye could be quantified by the UV track within the HPLC chromatogram. Up to 25% of native dye were accepted, since it has almost no influence on later radiochemical conversions. Traces of free excess chelator could not be quantified by the UV detector of the HPLC but by subsequent RCC with ^68^Ga. Here, we accepted RCC of more than 75%.

### Radiolabeling of [^68^Ga]Ga-NODA-GA-NHS-ester—Alexa Fluor™ 594 conjugate

It was crucial to ensure that all reactants were free from metal contaminants. All procedures involving radionuclides were performed manually while considering radiation protection. A suitable [^68^Ga]GaCl_3_ fraction in 0.6 M HCl was provided by the ^68^Ge/^68^Ga generator. Initial 0.6 ml of eluate were discarded due to insufficient activity. The following 0.6 ml were collected, and the activity (approximately 0.17 MBq/µl) was measured using the activimeter. The standard labeling procedure involved mixing 10 µl of [^68^Ga]GaCl_3_, 5 µl of ammonium acetate buffer (3 M, pH to 4.5), and 5 µl of the NODA-GA-NHS-ester—Alexa Fluor™ 594 conjugate. The optimized labeling was carried out in a 0.1 ml Eppendorf Fast PCR Tube for 5 min at room temperature (24 °C). Radiolabeling was confirmed via radio-HPLC analysis.

### Upscaling for animal experiments

To achieve high activities, two sequentially connected ^68^Ge/^68^Ga generators inline were used to elute [^68^Ga]GaCl_3_ with 0.6 M HCl. Initial 2 ml of eluate were discarded due to insufficient activity. The next 3 ml of eluate were concentrated and purified from residual ^68^Ge using a PS-H cartridge. The cartridge was briefly dried and [^68^Ga]GaCl_3_ was eluted from the cartridge in 0.1 ml fractions with 6 M ammonium acetate buffer (pH 4.5). Aliquots of the fifth fraction (0.1 ml, containing 40–50 MBq of ^68^Ga) were directly used in radiolabeling reactions.

The standard labeling procedure involved mixing 10 µl of ammonium acetate buffer (6 M, pH 4.5), 100 µl of [^68^Ga]GaCl_3_ and 15 µl of the NODA-GA-NHS-ester—Alexa Fluor™ 594 conjugate. The optimized labeling was carried out in a 0.1 ml Eppendorf Fast PCR Tube for 15 min at 80 °C. Radiolabeling was confirmed through radio-HPLC analysis. To remove unreacted [^68^Ga]GaCl_3_, the crude solution was passed through the C18 cartridge. In advance, the cartridge was conditioned with 2 ml of ethanol, followed by 5 ml of distilled water. The reaction solution was diluted with 5 ml of water and thus passed through the cartridge. The cartridge was washed with 7 ml of water, and the nonpolar product fraction was eluted with 0.5 ml of ethanol. This product was evaporated at 80 °C in a Argon gas stream. After cooling, the product was formulated in the desired solvent (either isotonic sodium chloride or phosphate-buffered saline).

## Data Availability

The datasets used and/or analysed during the current study are available from the corresponding author on reasonable request.

## Abbreviations

aSAH: Aneurysmal subarachnoid hemorrhage
PET: Positron emission tomography
^68^Ga: Gallium-68
NODA-GA-NHS ester: 2,2′-(7-(1-Carboxy-4-((2,5-dioxopyrrolidin-1-yl)oxy)-4-oxobutyl)-1,4,7-triazonane-1,4-diyl)diacetic acid
DMSO: Dimethyl Sulfoxide
MeCN: Acetonitrile
HPLC: High performance liquid chromatography
HCl: Hydrochloric acid
RCC: Radiochemical conversions
